# CIN coexisting with AIS is a risk factor for residual disease after conization for cervical adenocarcinoma *in situ*

**DOI:** 10.3389/fonc.2025.1571130

**Published:** 2025-06-09

**Authors:** Rong Zeng, Yulin Guo, Miao Zou, Chaonan Wang, Xufeng Wu

**Affiliations:** ^1^ Department of Gynecologic Oncology, Hubei Cancer Hospital, Tongji Medical College, Huazhong University of Science and Technology, Wuhan, China; ^2^ Cervical Cancer Control Center of Hubei Province. Maternal and Childe Health Hospital of Hubei Province, Wuhan, China

**Keywords:** AIS, AIS-plus-CIN, hysterectomy, residual disease, conization, negative margin

## Abstract

**Introduction:**

Compared to definitive hysterectomy, cervical conization with negative margin remains a controversial management for AIS currently. Our study aimed to evaluate (1) the effect of conization with negative margin alone or subsequent hysterectomy, (2) the effect of LEEP and CKC with or without subsequent hysterectomy, and (3) the correlation between clinicopathologic characteristics and residual disease after conization.

**Methods:**

This retrospective study involved 59 AIS patients who had negative margins through conization, with or without subsequent hysterectomy, focusing on clinicopathologic characteristics and outcomes.

**Results:**

A total of 59 patients with a median age of 34 years were followed for 3–95 months (median follow-up period: 35 months). Furthermore, 20 patients underwent subsequent hysterectomy (hysterectomy group) and 39 patients underwent conization alone (conization group). There were 24 patients who obtained negative margin through LEEP (LEEP group) and 35 patients through CKC (CKC group). Firstly, no significant difference in the rate of disease recurrence (2.6% vs. 0%, *P*-value = 1.0), HR-HPV positivity at first follow-up (15.4% vs. 5.0%, *P*-value = 0.404), or HR-HPV clearance (84.6% vs. 95.0%, *P*-value = 0.404) was found between the conization and hysterectomy groups. Secondly, no significant difference in the rate of disease recurrence (0% vs. 2.6%, *P*-value = 1.0), HR-HPV positivity at first follow-up (8.3% vs. 10.3%, *P*-value = 0.689), or HR-HPV clearance (91.7% vs. 89.7%, *P*-value = 0.689) was found between the CKC and LEEP groups. Lastly, five patients (25.0%) with residual disease were found in the hysterectomy group. All five patients were CIN coexisting with AIS (AIS-plus-CIN), and AIS-plus-CIN was identified as an independent risk for residual disease after conization (HR: 3.64, 95% CI: 1.01–4.26, *P*-value = 0.027). Moreover, one patient developing a recurrent disease was AIS-plus-CIN in the conization group.

**Discussion:**

Either CKC or LEEP with negative margin could achieve an equivalent effect compared with subsequent hysterectomy and could be recommended as an alternative therapy for AIS. However, subsequent hysterectomy is advised for AIS-plus-CIN.

## 1 Introduction

Adenocarcinoma *in situ* (AIS) is considered a precancerous lesion of adenocarcinoma of the cervix. Although it does not represent a common neoplasm, it has shown an apparent increase in incidence, especially in women of reproductive age (1). Compared with definitive hysterectomy, conization with negative margin was recommended as an acceptable therapy for the fertility-desiring AIS patients who are willing and able to adhere to surveillance. However, conization with negative margin remains a controversial management of AIS currently. There have been conflicting reports regarding the increase of disease recurrence, failure of HR-HPV (high risk-human papillomavirus) clearance, and incidence of residual disease in AIS patients undergoing conization (2). Firstly, to evaluate the equivalent effect of conization and hysterectomy, we analyzed the clinical outcomes including HR-HPV positivity, HR-HPV clearance, and disease recurrence of AIS patients treated by either conization with negative margin alone or subsequent hysterectomy in this study.

As conization can be performed using one of the following techniques—loop electrosurgical excision procedure (LEEP) or cold knife conization (CKC)—the optimal approach remains controversial, with the controversy focusing on effect and complications (3). To evaluate the equivalent effect of LEEP and CKC, we analyzed the HR-HPV positivity, HR-HPV clearance, and disease recurrence of AIS patients treated by CKC or LEEP (negative margin) with or without subsequent hysterectomy.

Lastly, the incidence of residual disease remains the main objection to conization with negative margin, which ranges from 0% to 44%, although a negative margin has a decreased risk of residual disease. Few research reported other risks related with residual disease, such as AIS-plus-CIN, multifocal disease, LEEP, and so on (4, 5). To achieve a better stratification of AIS patients who are optimal candidates for conization, we assessed residual disease in AIS patients who underwent subsequent hysterectomy for definitive surgery and investigated the correlation between certain features and residual disease after conization.

## 2 Materials and methods

### 2.1 Patients and data extraction

There was a total of 67 patients admitted to Hubei Cervical Cancer Prevention and Treatment Center between February 1, 2014 and September 30, 2019, all of whom had a diagnosis of AIS histologically by colposcopy-directed biopsy and cervical conization. Eight patients with positive margins were excluded, and 59 patients who had obtained negative margins were reviewed. This study analyzed the clinicopathological characteristics of patients retrospectively. Two cohort studies were conducted to compare the outcomes: (1) between conization with negative margin and hysterectomy and (2) between LEEP and CKC. The exclusion criteria were as follows: (1) conization specimen with positive margins, (2) pregnancy, and (3) lactation.

All cases were diagnosed through a three-step diagnostic procedure established by cytological screening and HR-HPV testing (step 1), colposcopy-directed biopsy (step 2), and conization pathology (step 3). Over 90% of the cytology, HR-HPV tests, and colposcopy-directed biopsy were performed at our institution, and the remainder was sourced from patients’ medical records (other medical institutions). Our institution performed all conization and hysterectomy surgeries. Study data were obtained following approval from the Ethics Review Committee of Hubei Maternal and Child Health Hospital.

### 2.2 HR-HPV and cytological testing

HR-HPV testing in our center was performed using the Cervista™ HPV HR test (Hologic, Inc., Marlborough, MA, USA), an *in vitro* diagnostic test for the detection of DNA from 14 types of HR-HPV (16, 18, 31, 33, 35, 39, 45, 51, 52, 56, 58, 59, 66, and 68), with results that were divided into A9, A7, and A5/6 groups, the Digene Hybrid Capture 2 test (Qiagen, Hilden, Germany), which detects 13 oncogenic genotypes (16, 18, 31, 33, 35, 39, 45, 51, 52, 56, 58, 59, and 68), with results classified as positive at a relative light unit value ≥1 pg/mL, and the Cobas 4800 test (Roche Molecular Systems, Pleasanton, CA, USA), which is able to detect the HPV16 and HPV18 genotypes separately as well as a group of hrHPV genotypes (HPV31, 33, 35, 39, 45, 51, 52, 56, 58, 59, 66, and 68). The Kaipu HPV 21 typing test (Guangzhou Kaipu Biotechnology Co., Ltd., Guangzhou, China), which classified HPV into 15 high-risk types (16, 18, 31, 33, 35, 39, 45, 51, 52, 53, 56, 58, 59, 66, and 68) and six low-risk types (6, 11, 42, 43, 44, and cp8304), was typically used for referred cases.

Cytology testing comprised liquid-based cytology testing using the ThinPrep^®^ 2000 system (Hologic, Inc.). Final cytological diagnosis was made using the Bethesda system (6, 7). Positive cytology findings included atypical squamous cells of unknown significance (ASC-US), atypical squamous cells, cannot exclude HSIL (ASC-H), low-grade squamous intraepithelial lesion (LSIL), high-grade squamous intraepithelial lesion (HSIL), and atypical glandular cells (AGC).

### 2.3 Clinical procedures and histological evaluation

**Figure 1:**
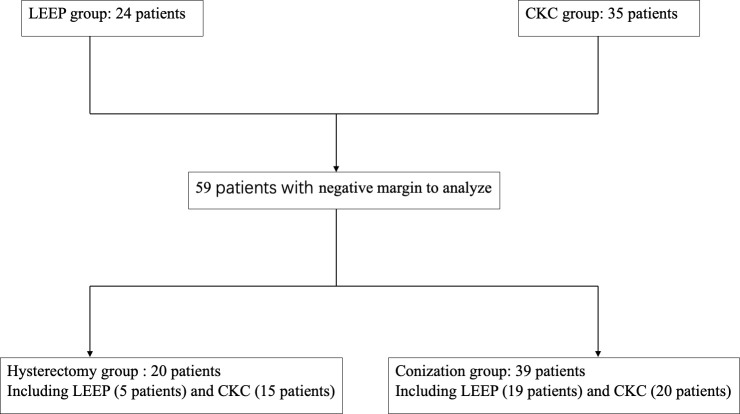
Breakdown of the 59 patients included and analyzed in the present study.

A total of 59 patients underwent primary conization, who had obtained negative margins, 35 patients underwent CKC (CKC group), and 24 patients underwent LEEP (LEEP group). Moreover, 20 (CKC: 15 cases; LEEP: five cases) of the 59 patients who had intention for definitive treatment underwent hysterectomy within a 2-week period following the conization (hysterectomy group), and the other 39 patients (CKC: 20 cases; LEEP: 19 cases) underwent conization alone (conization group). As shown in **Figure 1**, 59 patients were reviewed and divided into CKC, LEEP, hysterectomy, and conization groups.

Specimens of conization and hysterectomy were accurately evaluated at the pathology department of the hospital according to established criteria. AIS-plus-CIN (cervical intraepithelial neoplasia, CIN) was defined as both AIS and HSIL (CIN grade 2 or higher, HSIL) as confirmed by histopathology of either punch biopsy or conization (8). The margin status of conization was considered positive if any margin (ectocervical, endocervical, or deep/circumferential) was involved with either AIS or HSIL (CIN2/3), negative if all margins were histologically clear of *in situ* disease, and indeterminate if the margins could not be assessed or were not documented. Disease recurrence was defined as the presence of AIS or HSIL as confirmed by histology during follow-up. Residual disease was defined as the presence of either AIS or HSIL in specimens of subsequent hysterectomy.

### 2.4 Follow-up

All of the 59 patients were followed up for 3 to 95 months, and no one was lost to follow-up. After conization with a negative margin or hysterectomy, the patients were scheduled for regular follow-up at the outpatient clinic. Post-conization follow-ups were performed at 3, 6, 9, 12, 18, and 24 months and annually thereafter. Post-hysterectomy follow-ups were performed every 6 months for the first 2 years and then annually thereafter. Cervical or vaginal cytology and HR-HPV tests were performed at each visit, and patients with abnormal cytology findings and those who were HR- HPV-positive were referred for colposcopy-directed biopsy. HPV clearance was defined as a negative HR-HPV test at the first follow-up. All follow-up results were recorded.

### 2.5 Statistical analysis

Statistical analysis was performed using SPSS 24.0 software. The *t*-test was used for independent sample testing, and Cox regression analysis and χ^2^ test were used for statistical analysis of the data in this study. A *P*-value <0.05 was defined as statistically significant. A 95% confidence interval was used to calculate the risk degree. The average of measurement data was shown as the mean ± standard error of mean (SEM).

## 3 Results

### 3.1 Clinicopathological characteristics and clinical outcomes of patients

A total of 59 AIS patients with a median age of 34 years were reviewed in this study. Details of the clinicopathological characteristics including symptoms, histopathology, pre-treatment HR-HPV testing, and cytology are shown in **Table 1**. A definitive cytologic diagnosis of atypical glandular lesion (AGC) was made in four AIS patients (4/59, 6.8%), HR-HPV infection was detected in 54 patients (54/59, 91.2%), AIS-plus-CIN was diagnosed in 38 patients (38/59, 64.4%), multifocal disease was diagnosed in 16 patients (16/59, 27.1%), and none exhibited LVSI. The 59 patients underwent conization with negative margin, and 20 of the 59 patients underwent subsequent hysterectomy, whereas one patient developed a recurrence of AIS. The clinical outcomes including HPV positivity at first follow-up, HPV clearance, abnormal cytology during follow-up, and disease recurrence are presented in **Table 1** as well.

**Table 1:** The clinicopathological characteristics and clinical outcomes of 59 patients.

Clinicopathological characteristics	Cases: n/59(%)
Symptoms	
Symptom-free	40(67.8%)
Symptom-positive	19(32.2%)
Pre-treatment HR-HPV testing	
HPV16/18 positive	42(71.2%)
Other genotype HR-HPV positive	12 (20.3%)
Negative	5(8.5%)
Pre-treatment cytology	
NILM	18(30.5%)
Squamous intraepithelial lesion	36(61.2%)
AGC	5(8.5%)
Colposcopy-directed biopsy	
AIS	40/59(67.8%)
Histopathology	
AIS alone	21(35.6%)
AIS-plus-CIN	38(64.4%)
Multifocal disease	16(27.1%)
LVSI	0(0.0%)

Clinical outcomes	Cases: n/59(%)
Disease recurrence	1/59(1.7%)
HPV clearance	52/59(88.1%)
HPV positivity at first follow-up	7/59(11.9%)
Abnormal cytology during follow-up	2/59(3.4%)
ASCUS	1/59(1.7%)
HSIL	1/59(1.7%)

### 3.2 Therapeutic equivalence of conization with negative margin and definitive hysterectomy in AIS

A total of 20 of the 59 patients were willing to undergo subsequent hysterectomy (hysterectomy group) with a 2-week period following conization, and the other 39 patients treated by conization alone (conization group) underwent an immediate follow-up. The mean follow-up of the conization and hysterectomy groups was 32.4 ± 2.9 months (29.5–34.9 months, SEM: 2.9) and 39.7 ± 5.6 months (34.1–45.3 months, SEM: 5.6), respectively. During follow-up, only one patient of the conization group developed AIS recurrence. As shown in **Table 2**, between the abovementioned two groups, there was no significant difference in the rate of HPV positivity at first follow-up, HPV clearance, or disease recurrence (*P*-value >0.05). Additionally, there was no significant difference in clinicopathological characteristics, including mean age, mean follow-up period, rates of symptom-positive, pre-treatment HR-HPV infection, pre-treatment cytologic abnormality, AIS-plus-CIN, and multifocal disease (*P*-value >0.05).

**Table 2:** Comparison of the clinical outcomes and clinicopathological characteristics between Conization group (Conization-G) and Hysterectomy group (Hysterectomy-G).

Outcomes	Conization-G n/39 (%)	Hysterectomy-G n/20 (%)	*P*-Value
Disease recurrence	1 (2.6%)	0 (0.0%)	1.00
HPV clearance	33 (84.6%)	19 (95.0%)	0.404
HPV positivity at first follow-up	6 (15.4%)	1 (5.0%)	0.404

Clinicopathological characteristics	Conization-G n/39 (%)	Hysterectomy-G n/20 (%)	*P*-Value
Age	32.6±8.9yrs	41.3±5.9yrs	<0.001
Follow-up period	32.4±2.9 m	39.7±5.6 m	0.203
Symptom-positive	13 (33.3%)	6 (30.0%)	1.00
Pre-treatment HR-HPV infection	35 (89.7%)	19 (95.0%)	0.653
Pre-treatment cytologic abnormality	28 (71.8%)	13 (65.0%)	0.766
AIS-plus-CIN	28 (71.8%)	10 (50.0%)	0.091
Multifocal disease	12 (30.8%)	4 (20.0%)	0.539

### 3.3 Therapeutic equivalence of LEEP and CKC with negative margin

Out of the 59 patients who had obtained negative margins, 24 patients underwent LEEP (LEEP group), and 35 patients underwent CKC (CKC group). As shown in **Table 3**, between the abovementioned two groups, there was no significant difference in the rate of HPV positivity at first follow-up, HPV clearance, or disease recurrence (*P*-value >0.05). Additionally, there was no significant difference in clinicopathological characteristics, including mean age, mean follow-up period, and the rate of symptom-positive, pre-treatment HR-HPV infection, pre-treatment cytologic abnormality, AIS-plus-CIN, multifocal disease, and subsequent hysterectomy (*P*-value >0.05).

**Table 3:** Comparison of the clinical outcomes and clinicopathological characteristics between LEEP and CKC group.

Outcomes	LEEP group n/24 (%)	CKC group n/35 (%)	*P*-Value
Disease recurrence	0 (0.0%)	1 (2.6%)	1.000
HPV clearance	22 (91.7%)	30 (85.7%)	0.689
HPV positivity at first follow-up	2 (8.3%)	5 (14.3%)	0.689

Clinicopathological characteristics	LEEP group n/24 (%)	CKC group n/35 (%)	*P*-Value
Age	34.9±1.8yrs	36.0±1.6yrs	0.645
Follow-up period	34.7±4.3 m	34.9±3.6 m	0.962
Symptom-positive	9 (37.5%)	10 (28.6%)	0.574
Pre-treatment HR-HPV infection	23 (95.8%)	31 (88.6%)	0.630
Pre-treatment cytologic abnormality	17 (70.8%)	24 (61.5%)	1.000
AIS-plus-CIN	13 (54.2%)	25 (71.4%)	0.268
Multifocal disease	8 (33.3%)	8 (22.9%)	0.390
Subsequent hysterectomy	5 (20.8%)	15 (42.9%)	0.099

### 3.4 Higher likelihood of residual and recurrent disease in AIS-plus-CIN patients, after conization with negative margin

In the hysterectomy group, five of 20 patients had a residual disease (5/20, 25.0%, residual disease group); the other 15 patients had no residual disease (non-residual disease group), and all of the five patients were AIS-plus-CIN. To explore the risks related to residual disease after conization with negative margin, Cox regression was used to analyze the correlation between certain features and residual disease in the hysterectomy group. The features of the residual disease group and the non-residual disease analyzed through Cox regression are listed in **Table 4**, including age, follow-up period, symptom-positive, pre-treatment cytologic abnormality, LEEP, AIS-plus-CIN, multifocal disease, and HPV clearance.

**Table 4:** The features of Residual disease and Non-residual disease groups analyzed through Cox regression.

Features	Residual group n/5 (%)	Non-residual group n/15 (%)
Age	40.2±2.4yrs	41.7±1.6yrs
Follow-up period	53.2±7.6m	35.2±6.7m
Symptom-positive	1/5(20.0%)	5/15(33.3%)
Pre-treatment cytologic abnormality	3/5(60.0%)	8/15(53.3%)
LEEP	0/5(0%)	5/15(33.3%)
AIS-plus-CIN	5/5(100.0%)	5/15(33.3%)
Multifocal disease	2/5(40.0%)	2/15(13.3%)
HPV-clearance	5/5(100.0%)	14/15(93.3%)

**Figure 2:**
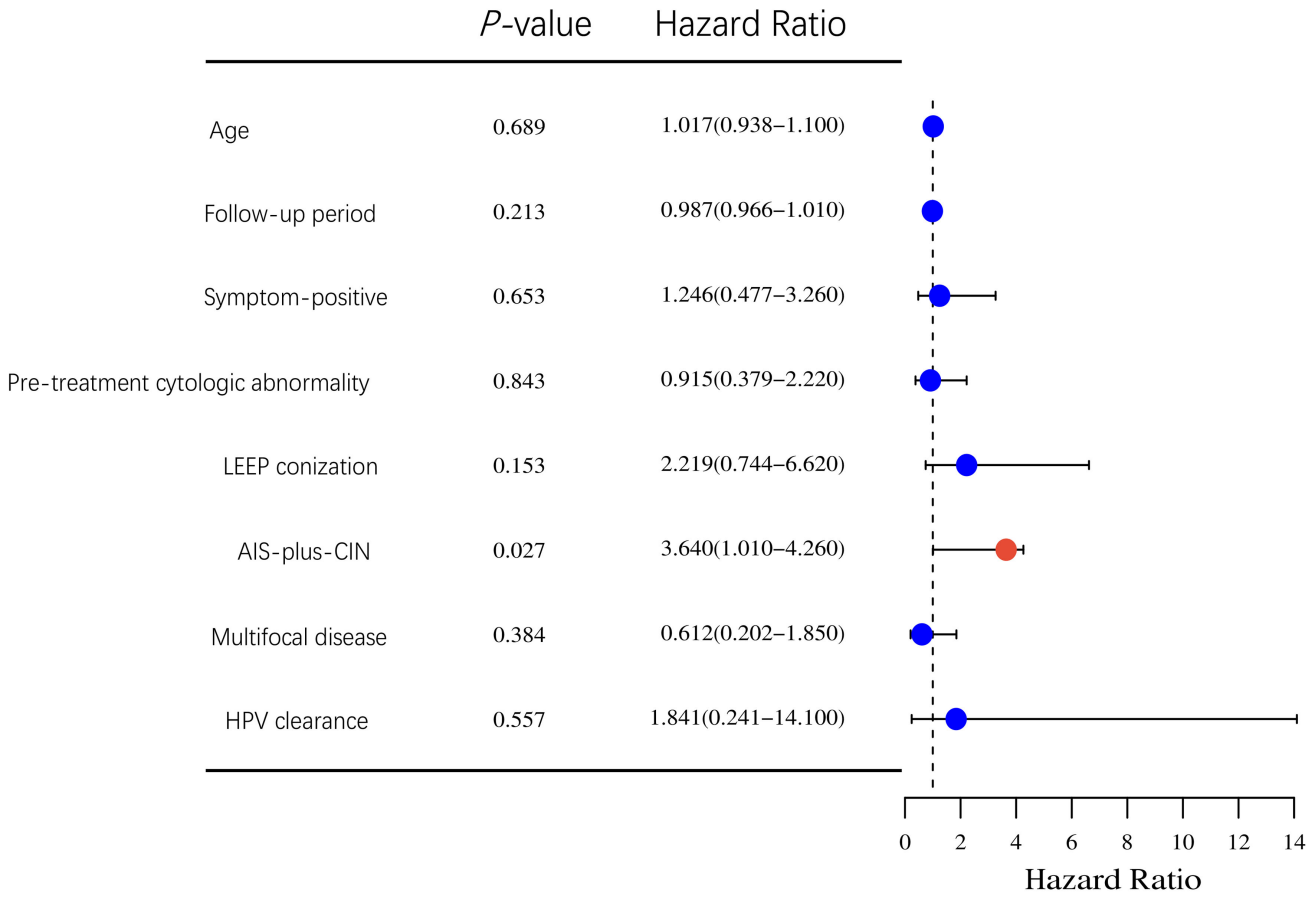
Cox regression analysis of correlation between clinicopathological characteristics and residual disease in the hysterectomy group.

As shown in **Figure 2**, AIS-plus-CIN (HR 3.64, 95% CI: 1.01–4.26, *P*-value = 0.027), as the only one independent risk for residual disease, was found. Other factors were not associated with residual disease. Notably, no patient with residual disease was from the LEEP group; however, Cox regression in this study showed that LEEP was not associated with residual disease. Interestingly, in our study, only one patient was found to develop a recurrent disease, and that only one patient was also AIS-plus-CIN.

## 4 Discussion

### 4.1 Discussion on the clinicopathologic characteristics of AIS

#### 4.1.1 AIS usually occurs in women of reproductive age; fertility-sparing surgery should be considered

A meta-analysis including 1,278 cases of AIS reported that the average age at AIS diagnosis was 36.9 years; most cases occurred between the ages 32 and 40, with almost 70% of the cases affecting women less than 35 years (9). Similarly, the median age of AIS patients in our study was 34 years. Cervical conization, a fertility-sparing surgery, should be considered for women with AIS who are of child-bearing age.

#### 4.1.2 HR-HPV infection was associated with the development and prognosis of AIS

The same with cervical adenocarcinoma, HR-HPV infection is associated with the development of AIS. In the past 15 years, research revealed that the rate of HR-HPV infection was 56.2%–93.0% in AIS, while HPV16 and HPV18 were the most common types, with the infection rate ranging from 79% to 96% (10, 11). Also, the rate of HR-HPV infection was 91.5%, with a HPV16/18 infection rate of 71.2% in our study. Furthermore, it has been observed that HR-HPV persistence closely associates with AIS recurrence. Therefore, HPV testing is recommended as a screening and follow-up method for AIS, and HPV clearance is considered as an indicator of treatment effect in AIS (12). Our study likewise investigated the rate of HPV clearance as one of the indicators of outcomes.

#### 4.1.3 The accuracy of cervical cytology and colposcopy-directed biopsy for the diagnosis of AIS was relatively low

This low accuracy was due to sampling difficulties from the concealed location of AIS above the squamocolumnar junction and within the endocervical canal as well as the challenges in recognizing AIS because of subtle cytological features that overlap with neoplastic squamous and non-neoplastic endocervical and endometrial cells. Previous research indicated that the detection rate of overall cytological abnormalities in AIS patients (including squamous epithelial abnormalities) was 58.0% to 92.0%, with AGC accounting for 4.4% to 33.4% (13). The diagnostic accuracy of colposcopy-directed biopsy ranged from 30% to 50% (14). In our study, we detected cytological abnormalities at a rate of 69.7%, with AGC accounting for 8.5%. Our study reconfirmed the low accuracy of cervical cytology for diagnosing AIS, consistent with findings reported in the literature. A total of 40 cases were diagnosed through colposcopy-directed biopsy, achieving a diagnostic accuracy of 67.8% (40 out of 59), which is significantly higher than those reported in previous studies (30%–50%). This improved diagnostic accuracy of colposcopy-directed biopsy was a result of the rigorous training of colposcopy operators at our center. With a diagnostic accuracy approaching 70%, colposcopy-directed biopsy, when combined with HPV and cytological testing, presented a practical strategy for the screening and follow-up of AIS in this study.

#### 4.1.4 Multifocal disease caused by “skip lesions” was more likely to occur in AIS

Multifocal disease was significantly more prevalent in AIS than in CIN primarily due to AIS’ histological origin, which featured “skip lesions” as a form of metastasis. Multifocal disease in AIS, with the occurrence rate ranging from 10% to 15% and with outbreaks separated by at least 2 mm of normal mucosa, was reported by Duncan. In our study, multifocal disease with the occurrence rate of 27.1% (16/59) was confirmed through histopathology, even higher than the rate reported in the literature. This increase was attributed to the stringent guidelines regarding the extent of conization excision at our center, which required a cone base diameter of at least 2 cm and a cone height of at least 1.5 cm.

The clinicopathologic characteristics of AIS created a significant controversy around cervical conization with negative margin, which is considered an alternative treatment to hysterectomy for preserving fertility. Conization was believed to have several inherent disadvantages compared to hysterectomy, namely: (1) the low diagnostic accuracy of cytology and colposcopy-directed biopsy complicates AIS monitoring during follow-up. However, our study reported a diagnostic accuracy of up to 70% through colposcopy-directed biopsy, combined with HR-HPV and cytology testing, which could mitigate this issue; (2) the concealed location of AIS complicates the complete excision of the lesion by conization. Thus, CKC was regarded as the “gold standard” while LEEP remains a controversial alternative; and (3) multifocal disease in AIS raises the risk of recurrent disease, HPV clearance failure, and residual disease.

### 4.2 Discussion on the treatment of AIS

#### 4.2.1 Compared to hysterectomy, cervical conization with negative margin had non-inferiority in effect

Recurrent disease and cervical adenocarcinoma were observed in 7.4% and 1.9%, respectively, among patients who underwent cervical conization, and post-conization HR-HPV positivity was confirmed as a valid predictor of disease relapse and progression, leading to hysterectomy being established as the standard definitive treatment for AIS (15). Currently, conflicting reports about the recurrent disease in AIS undergoing conization existed, and the debate between conization and hysterectomy is still going on. Our study assessed the difference of clinical outcomes between the conization group and the hysterectomy group, and it showed that there was no significant difference in the rate of disease recurrence or HPV clearance, suggesting that cervical conization with negative margin could achieve an effect equivalent to that of hysterectomy. Our findings aligned with the conclusions drawn by Costa and Baalbergen (16, 17).

#### 4.2.2 Compared to CKC, LEEP with a negative margin has non-inferiority in effect

Compared to CKC, LEEP offered advantages including the ability for the procedure to be performed in an outpatient setting, lower morbidity, and fewer adverse obstetric outcomes. It also offered disadvantages including incomplete excision induced by smaller excision and significant thermal artefact (18). Two systematic reviews have reported higher rates of incomplete excision with LEEP at 44%–51% compared to 29%–30% with CKC. They concluded that LEEP was safe and comparable to CKC when negative margins were achieved (19, 20). In our study, the margins of all the excisions by LEEP could be evaluated as well as CKC, and no difference in disease recurrence or HPV clearance was found between the CKC and LEEP groups. In addition, none of the patients in the LEEP group who underwent subsequent hysterectomy had a residual disease. These results were consistent with the observations of a phase II pilot randomized controlled trial, which demonstrated that LEEP did not result in a higher incidence of positive margins or smaller cone excisions compared to CKC (21).

In a word, it was suggested that either LEEP or CKC with negative margin can achieve the equivalent therapeutic effect compared to subsequent hysterectomy in AIS.

#### 4.2.3 Even when negative margins had been obtained by conization, subsequent hysterectomy was recommended for patients with AIS-plus-CIN

The threat of a residual disease after cervical conization was inevitable. Previous studies have reported residual disease rates of 0%–44% (18), and a residual disease was also found in five of 20 patients (25%) in the hysterectomy group. The risks related to residual disease were further analyzed in our study. We found that AIS-plus-CIN is an independent risk factor for residual disease in AIS patients who have undergone conization with a negative margin. The same result has also been reported by Song and Codde (22, 23). Moreover, a survey on AIS in the US showed that up to 50.8% of cases involved AIS-plus-CIN, which was related to poor prognosis (9). In our study, 38 AIS-plus-CIN patients were identified with an even higher proportion, accounting for 64.4%. Notably, the only patient who had a recurrence during follow-up was also AIS-plus-CIN. This suggested that subsequent hysterectomy could be the optimal therapy for AIS-plus-CIN patients.

In conclusion, hysterectomy is advised for patients diagnosed with AIS-plus-CIN due to the correlation between residual disease and AIS-plus-CIN. Our findings indicated that LEEP or CKC with a negative margin could achieve an equivalent effect compared to a subsequent hysterectomy. Thus, LEEP or CKC with a negative margin could be recommended as an alternative therapy for AIS alone.

## Data Availability

All data generated or analyzed during this study are included in this published article.
