# Correction: CIN coexisting with AIS is a risk factor for residual disease after conization for cervical adenocarcinoma *in situ*

**DOI:** 10.3389/fonc.2025.1649631

**Published:** 2025-07-18

**Authors:** Rong Zeng, Yulin Guo, Miao Zou, Chaonan Wang, Xufeng Wu

**Affiliations:** ^1^ Department of Gynecologic Oncology, Hubei Cancer Hospital, Tongji Medical College, Huazhong University of Science and Technology, Wuhan, China; ^2^ Cervical Cancer Control Center of Hubei Province. Maternal and Child Health Hospital of Hubei Province, Wuhan, China

**Keywords:** AIS, AIS-plus-CIN, hysterectomy, residual disease, conization, negative margin

In the published article, there was an error in Affiliation 1. Instead of “Department of Gynecologic Oncology, Hubei Cancer Hospital, Wuhan, China”, it should be “Department of Gynecologic Oncology, Hubei Cancer Hospital, Tongji Medical College, Huazhong University of Science and Technology, Wuhan, China”.

The original version of this article has been updated.

